# Autophagy induction causes a synthetic lethal sensitization to ribonucleotide reductase inhibition in breast cancer cells

**DOI:** 10.18632/oncotarget.6539

**Published:** 2015-12-09

**Authors:** Yun-Ru Chen, Brittany Tsou, Shuya Hu, Huimin Ma, Xiyong Liu, Yun Yen, David K. Ann

**Affiliations:** ^1^ Department of Molecular Pharmacology, Beckman Research Institute, City of Hope National Medical Center, Duarte, CA, USA; ^2^ Department of Diabetes and Metabolic Research, Beckman Research Institute, City of Hope National Medical Center, Duarte, CA, USA

**Keywords:** autophagy, ribonucleotide reductase, synthetic lethality, breast cancer, tamoxifen

## Abstract

Macroautophagy can promote cellular survival or death depending on the cellular context and its extent. We hypothesized that autophagy induction would synergize with a therapeutic agent targeting the autophagic cargo. To test this hypothesis, we treated breast cancer MDA-MB-231 cells with tamoxifen (TMX), which induces autophagy through an estrogen receptor-independent pathway. Induction of autophagy reduced cellular levels of RRM2, a subunit of ribonucleotide reductase (RR), the rate limiting enzyme in the production of deoxyribonucleotide triphosphates (dNTPs). This autophagy inducer was combined with COH29, an inhibitor developed in our laboratory that targets RR through a novel mechanism. The combination therapy showed synergistic effects on cytotoxicity *in vitro* and in an *in vivo* xenograft model. This cytotoxicity was blocked by knockdown of the autophagy protein ATG5 or addition of chloroquine, an autophagy inhibitor. The combined therapy also induced dNTP depletion and massive genomic instability, leading us to hypothesize that combining autophagy induction with RR inhibition can lead to mitotic catastrophe in rapidly dividing cells. We propose that this TMX + COH29 combined therapy may have clinical benefit. Furthermore, autophagy induction may be a general mechanism for augmenting the effects of chemotherapeutic agents

## INTRODUCTION

Cell death is an important process in cancer therapy that occurs through three major pathways: apoptosis, autophagy, and necrosis - each of which can be characterized by various morphological criteria [1]. First, apoptosis is a form of programmed cell death that involves cell rounding, DNA fragmentation, and cell membrane blebbing. Subsequent engulfment of apoptotic cells by phagocytes prevents an inflammatory response to the dead cells. Second, autophagy is a self-degradative pathway for disposal of obsolete parts of the cell, is accompanied by vacuolization in the cytoplasm and can lead to increased cell survival or cell death depending on the type and extent of self-degradation [2]. During autophagy, organelles and parts of the cytoplasm are isolated in characteristic double-membrane organelles known as autophagosomes. Fusion of the autophagosomes with lysosomes can allow for degradation and recycling as part of the quality control process that maintains bioenergetics and basic cell functions [3, 4]. However, we and others have also shown that prolonged starvation of arginine induces oxidative stress and triggers autophagic cancer cell death [5, 6]. Third, necrosis is defined as a type of cell death that lacks features of the two previous pathways and is accompanied by swelling of the cell, loss of membrane integrity, and inflammatory response, as the cellular contents are released into the extracellular environment [1]. Cell death pathways play important roles in tumorigenesis, cancer progression, and resistance to therapy and thus are important therapeutic targets in cancer [7].

One therapeutic approach is to induce an imbalance of critical cellular components, leading to reduced proliferation and death of the cancer cells. For instance, deoxyribonucleotide triphosphates (dNTP) are required building blocks for cells to carry out the basic functions of DNA synthesis and repair. Thus, imbalanced levels of dNTPs result in mutagenesis, genomic instability and cell death [8]. The intracellular supply of dNTPs is regulated by ribonucleotide reductase (RR), an enzyme that catalyzes the formation of deoxyribonucleotides from ribonucleotides, and plays a critical role in regulating the total rate of DNA synthesis [9]. RR is also one of the top 50 metabolic enzymes frequently overexpressed in human tumors [10]. As a result, RR is a popular target for anticancer agents such as RR inhibitors [11]. RR consists of two protein subunits, RRM1 and RRM2, both of which are required for enzymatic activity. Two RRM1 subunits and two RRM2 subunits join together to form the active tetrameric enzyme. RRM1 and RRM2 are encoded by different genes on separate chromosomes and, most importantly, their messenger RNAs (mRNAs) are differentially expressed throughout the cell cycle. The cellular RRM1 protein level remains relatively stable through the entire cell cycle and has a relatively long half-life of 18 to 24 h. In contrast, RRM2 is only expressed during the late G_1_-/early S-phase when DNA replication occurs, has a short half-life of 3 to 4 h, and is rapidly degraded in late mitosis. Therefore, RR enzyme activity is largely determined by the RRM2 protein levels in normal cells.

We have identified a novel small molecule RR inhibitor, COH29 that disrupts the interactions between the hRRM1 and RRM2 subunits of human RR [12]. COH29 is an aromatically substituted thiazole compound [N-(4-(3,4-dihyrophenyl)-5-phenylthiazol-2-yl) -3,4-dihydroxybenzamide], which we developed from a virtual screen of the National Cancer Institute (NCI) Diversity Set with 2000 compounds (NCI2000). We screened for the ability to bind a pocket on the surface of the M2 subunit of RR, as predicted by protein structural analysis [12]. Candidate compounds identified from this screening were further optimized by iterative virtual and structure activity relationship (SAR) analysis to yield COH29. The aromatically substituted thiazole compound binds to RRM2 and occupies a ligand-binding pocket on hRRM2 that is positioned close to the hRRM1-hRRM2 interface. It thereby impedes formation of the holoenzyme complex and inhibits RR activity. Unlike other RR inhibitors, COH29 does not appear to chelate iron, so side effects are likely to be reduced. Through interference with DNA repair and replication pathways, COH29 upsets the balance of dNTP in cells, leading to cell death. Because COH29 has limited effects as a single therapeutic agent, we used various biochemical, cell biological, and xenograft approaches to find a combination therapy that reduced the toxicity and increased the efficacy of COH29.

Tamoxifen (TMX), a nonsteroidal estrogen-receptor α (ERα) modulator, is a widely used therapeutic agent for patients with ERα-positive breast cancers [13, 14]. TMX primarily functions by competing with estrogen for binding to ERα, thereby inhibiting ERα activity [15]. As a result, TMX is primarily used to reduce the recurrence of ERα-positive breast tumors [16]. However, results from both pre-clinical and clinical investigations have demonstrated a beneficial effect of TMX on ERα-negative tumors or cancer cells [13, 17-21], suggesting that TMX can also act in an ERα-independent manner. Along the same lines, *in vitro* studies have suggested that TMX, besides inducing apoptosis in tumor cells, also promotes the accumulation of large-scale autophagic structures. These findings indicate that TMX may induce cancer cell death through autophagy [22, 23]. The role of autophagy in cancer cell death has been exploited by both pharmacological and genetic approaches that modulate autophagy [3, 24, 25]. Modulating autophagy requires extra caution because autophagy can protect cancer cells in the early stages of chemotherapy, but promote tumor cell death afterwards [3, 24, 25]. Furthermore, autophagy can be induced by various stimuli, including nutrient deprivation, serum starvation, metabolic stress, radiation, and anticancer drugs in multiple cancer cells [24, 26-29]. We propose to exploit autophagy-dependent cell death to promote the efficacy of COH29-based cancer therapies.

Our previous study has shown that the regulation of the dNTP pool and RRM2 levels are inversely related to levels of autophagy [30]. In that report, reduced RRM2 abundance was associated with, increased autophagy and a decreased intracellular dNTP pool. On the other hand, overexpression of RRM2 or supplementation of exogenous deoxyribonucleotide monophosphates (dNMPs) to increase the intracellular dNTP levels attenuated autophagy [30]. We hypothesized that adding an autophagy inducer may boost the effectiveness of COH29 by reducing the total levels of RRM2. Therefore, we evaluated the effects of combining TMX with COH29 in ER-negative MDA-MB-231 breast cancer cells both *in vitro* and *in vivo*. We found that combined use of TMX and COH29 reduced intracellular dNTP levels, induced genomic instability, promoted tumor cell death, and reduced tumor volume in a xenograft model. These effects were dependent on autophagy. The effects of the drug combination were far greater than the effects of either drug alone. This study proposes an effective anti-tumor regimen and, more broadly, establishes aggressive autophagy induction as a plausible method for increasing the sensitivity of cells to chemotherapy.

## RESULTS

### Tamoxifen sensitizes breast cancer MDA-MB-231 cells to COH29 through autophagy induction

**Figure 1:**
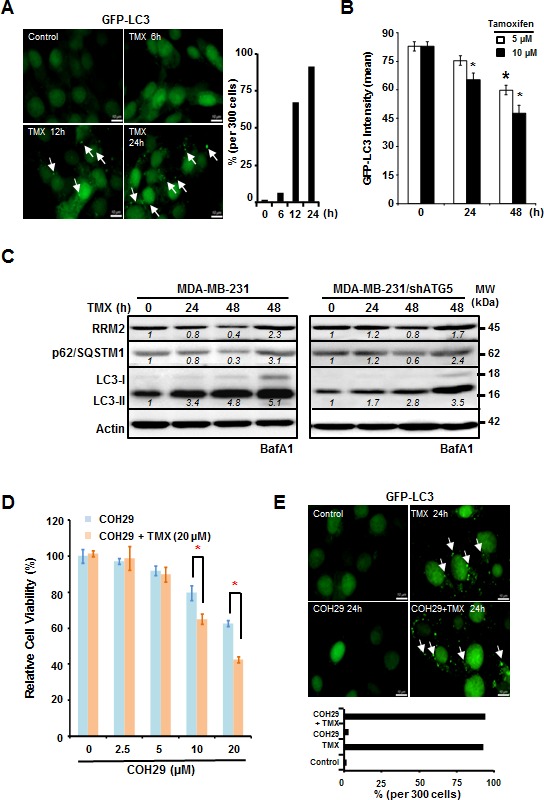
Tamoxifen enhances the cytotoxic effects of COH29 through autophagic degradation of RRM2 **A.** and **B.** Tamoxifen (TMX) induces autophagy in MDA-MB-231 cells. MDA-MB-231 cells with stable integration of GFP-LC3 were treated with TMX (5 or 10 μM) for the indicated time periods and analyzed with microscopy **A.** and flow cytometry **B.**. **A.** Arrows indicate GFP-LC3 puncta. The percentage of cells containing GFP-LC3 puncta was enumerated by counting 300 cells and plotted (*right panel*). **B.** For flow cytometry, quantitation was showed in means ± SD from three independent experiments; _*_: *p* < 0.05 versus control. **C.** Knockdown of ATG5 stabilizes RRM2 in TMX-treated MDA-MB-231 cells. Western blotting was performed to determine RRM2 abundance, p62/SQSTM1 degradation, and LC3 lipidation (LC3-II) in autophagy-competent MDA-MB-231 and autophagy-impaired MDA-MB-231/shATG5 cells. Actin abundance was used as an internal control to ensure that equal amounts of proteins were loaded in each lane. Bafilomycin A1 (BafA1; 10 nM) was used to validate the lysosomal degradation. One representative Western from *n* = 3 is shown. **D.** TMX sensitizes MDA-MB-231 cells to COH29. MDA-MB-231 cells were cultured with increasing concentrations of COH29 with and without 20 μM TMX for indicated time periods prior to cytotoxicity assays. The means ± SD from three independent experiments are shown; _*_: *p* < 0.05 versus control. **E.** *COH29 does not affect the induction of autophagy by TMX*. Arrows indicate GFP-LC3 puncta. (A, D) Scale bar: 10 μm.

Based on our previous work [30], we hypothesized that inducing autophagy would decrease RRM2 levels and sensitize cancer cells to COH29, a novel RR inhibitor [12, 31]. Consequently, we combined TMX, an autophagy inducer, with COH29 in ERα-negative (ER-) breast cancer cells and measured the resulting cytotoxicity [32, 33]. We transfected the cells with green fluorescent protein (GFP) linked to the autophagosome marker microtubule associated protein 1A/1B light chain (LC3). TMX treatment induced autophagy in MDA-MB-231 cells (Figure 1A, left panel), as evidenced by the accumulation of GFP-LC3 puncta over time (Figure 1A, right panel). Addition of TMX (10 μM) to the MDA-MB-231/GFP-LC3 cells caused a significant decrease in GFP intensity starting at 24 h post-treatment, whereas treatment with a lower concentration of TMX (5 μM) needed up to 48 h to render a significant decrease (Figure 1B). These results suggested that TMX treatment induces autophagy in MDA-MB-231 cells in a concentration- and time-dependent manner.

Western analyses were used to monitor the protein abundances of RRM2, LC3 and p62/SQSTM1, another autophagy marker, in breast cancer cells treated with TMX for 24 and 48 h. Consistently, TMX decreased RRM2 abundance in several breast cancer cell lines including MDA-MB-231, MCF7 and T47D, regardless of their ERα status (Figure 1C and Supplementary Figure S1A). However, TMX had no effect on RRM2 abundance in autophagy-deficient ATG5-knockdown MDA-MB-231 (MDA-MB-231/shATG5) cells (Figure 1C). We also used western analysis to measure the autophagy-induced lipidation of LC3. As expected, LC3 lipidation increased (appearance of LC3-II) and p62/SQSTM1 abundance decreased in response to TMX, but accumulated in the presence of bafilomycin A1 (BafA1), a potent and selective inhibitor of vacuolar H^+^-ATPase (V-ATPase) that prevents the autophagic degradation via lysosomes [34, 35]. This result further supports a model where TMX induces autophagy. The effect of BafA1 observed in MDA-MB-231/shATG5 was due to the incomplete knockdown of ATG5 (Figure 1C, right panel).

Next, we hypothesized that the TMX-induced, autophagy-mediated RRM2 downregulation sensitizes MDA-MB-231 cells to anti-RR agents, such as COH29. To test this possibility, MDA-MB-231 cells were maintained with the increasing concentrations of COH29 -in the presence or absence of TMX for 72 h. Cell viability was determined using acid phosphatase (ACP) assays at 72 h post-treatment (Figure 1D). As expected, the combination treatment was more effective at reducing cell viability than COH29 alone. The IC50 for COH29 is approximately 35 μM and 17 μM in the presence and absence of TMX, respectively (Figure 1D). We further confirmed that the combination treatment also resulted in autophagy induction by analyzing GFP-LC3 puncta formation (Figure 1E, upper panel). The combined treatment with COH29 did not affect the ability of TMX to induce autophagy (Figure 1E, lower panel). Moreover, TMX induced RRM2 reduction was also observed in the ERα-positive breast cancer T47D and MCF7 cells (Supplementary Figure S1A), suggesting that it is not breast cancer subtype is specific. Along the same line, a dose-dependent cytotoxic effect by the combination of COH29 and TMX was also noted in T47D and MCF7 cells (Supplementary Figure S1B). However, the non-tumorigenic mammary MCF-10A cells were less sensitive to the combined treatment (Supplementary Figure S1B). We have previously shown that rapamycin decreased RRM2 level in an autophagy-dependent manner in KB cells [30]. Here, we further demonstrated that rapamycin (10 μM) also sensitized MDA-MB-231 cells to COH29 treatment by reducing RRM2 expression (Supplementary Figures S2A and S2B). The involvement of autophagy in promoting COH29 cytotoxicity was further examined in the context of arginine deprivation with arginine deiminase (ADI, 0.2 μg/ml), known to induce rapamycin-independent autophagy [6], and comparable results were observed (Supplementary Figures S2C and S2D). Together, these results support a model where autophagy induction, through TMX, sensitizes cancer cells to COH29-induced cell death.

### The synergistic effects of COH29 + tamoxifen combination are autophagy-dependent

**Figure 2:**
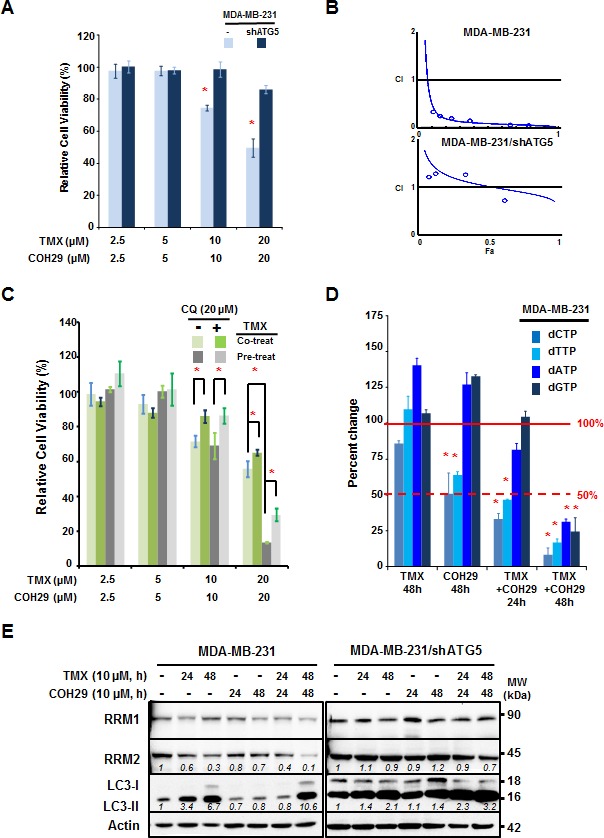
Autophagy is required for the synergistic effect between tamoxifen and COH29 **A.** Knockdown of ATG5 abolishes the synergistic cytotoxicity of the TMX + COH29 combination. MDA-MB-231 and MDA-MB-231/shATG5 cells were plated for the presence of indicated treatments. Cell viability was determined using the acid phosphatase assay at 72 h post-combined treatment and significance was determined relative to the no treatment control. Shown are the means ± SD; *n* = 3; _*:_ *p* < 0.05. **B.** Synergistic effect of COH29 combined with TMX. Cytotoxicity data were evaluated for the combination index (CI) using CompuSyn software. The line shown in the plot indicates CI = 1 and values below 1 are defined as synergistic effects. *n* = 3. **C.** Pre-treatment with TMX improves the effect of COH29. MDA-MB-231 cells were pre-treated with vehicle or TMX for 24 h prior to exposure to TMX and COH29, as indicated, for 72 h. Chloroquine (CQ, 20 μM) addition significantly reversed the cytotoxic effects of COH29 and TMX. Results are shown as means ± SD; *n* = 3; _*:_ *p* < 0.05. **D.** Combination of TMX and COH29 decreases the intracellular dNTP pool. MDA-MB-231 cells were treated as indicated prior to cell harvesting. The intracellular abundance of dNTPs including dCTP, dTTP, dATP and dGTP was determined and the relative fold change in cells subjected to the indicated treatment was calculated by comparison to the no-treatment control. Results are shown as means ± SD; *n* = 3; _*:_ *p* < 0.05. The solid line indicates 100% and the dashed line shows a 50% decrease. **E.** Autophagy is required to decrease RRM2 abundance. MDA-MB-231 and MDA-MB-231/shATG5 cells were subjected to treatment as indicated, followed by western analysis. One representative western blot from *n* = 3 is shown.

To explore the mechanism underlying the improved efficacy of combining TMX with COH29, MDA-MB-231 and MDA-MB-231/shATG5 cells were treated with the combination of COH29 and TMX. Figure 2A reveals that MDA-MB-231 cells were more sensitive to treatment than their isogenic autophagy-deficient (ATG5-knockdown) counterparts, suggesting that the combinatorial effect of TMX and COH29 was autophagy-dependent (Figure 2A). The viability data was further analyzed by Compusyn software to determine whether the drugs showed synergistic effects. The combination index (CI) for the combination in MDA-MB-231 was 0.18, indicating a synergistic effect, whereas the CI was 1.53 in MDA-MB-231/shATG5 cells, suggesting no evidence of synergy (Figure 2B). To further confirm the synergistic effect is autophagy-dependent, knockdown of BECLIN1 or ATG5, a key molecule for autophagy induction, also conferred a pro-survival effect, like in the context of combined treatment (Supplementary Figure S1C). Next we determined if the combined inhibitory effect was autophagy-dependent by using an autophagy inhibitor, chloroquine (CQ). The cells were treated with TMX and COH29, as indicated, in the presence and absence of CQ (20 μM). The addition of CQ resulted in a significant rescue of cell viability in cells treated with 10 or 20 μM each of TMX and COH29 (Figure 2C). Moreover, pre-treatment with TMX for 24 h to induce autophagy notably enhanced the cytotoxicity induced by TMX and COH29 (20 μM each). Consistently, the enhanced cytotoxic effect of sequential treatment was also retarded by CQ addition (Figure 2C).

Next, we tested the hypothesis that reduced RRM2 levels would deplete intracellular dNTP pools. We measured the dNTP pools in treated cells, and found that COH29 alone significantly decreased dCTP and dTTP abundance to 50% at 48 h post-treatment; however, the combined treatment showed a comparable effect at 24h (Figure 2D). At 48 h after the combined treatment, all of the dNTP pools, including dCTP, dTTP, dATP and dGTP, were reduced to less than 25% of the control (Figure 2D). Likewise, Western analyses revealed that TMX-induced LC3-II accumulation and RRM2 reduction were retarded by knocking-down ATG5 (Figure 2E). Although, the expression of RRM1 was not affected, the abundance of RRM2 is more reduced by the combination of TMX and COH, which was consistent with the cytotoxicity data shown in Figure 2A. These results support our overall hypothesis that decreasing RR level decreases intracellular dNTP abundance.

Lastly, we investigated the possibility that other cell death pathways were activated by the combined treatment. To study the contribution of apoptosis to the cell death, MDAMB231 and MDAMB231/shATG5 cells were treated with a combination of TMX and COH29 for 24 and 48 h. The percentage of Annexin V-positive (early apoptotic) and/or PI-positive (dead) cells was determined by flow cytometry. The low percentage of cells undergoing early apoptosis suggested that apoptosis could not completely account for the observed death (Supplementary Figure S3A). Western analyses further supported this finding by showing that the majority of caspase 3 remained inactivated (not cleaved) (Supplementary Figure S3B). Taken together, our results support the conclusion that the combined use of TMX and COH promoted cytotoxicity by autophagy induction.

### Reduced *in vivo* tumor progression after tamoxifen and COH29 administration

**Figure 3:**
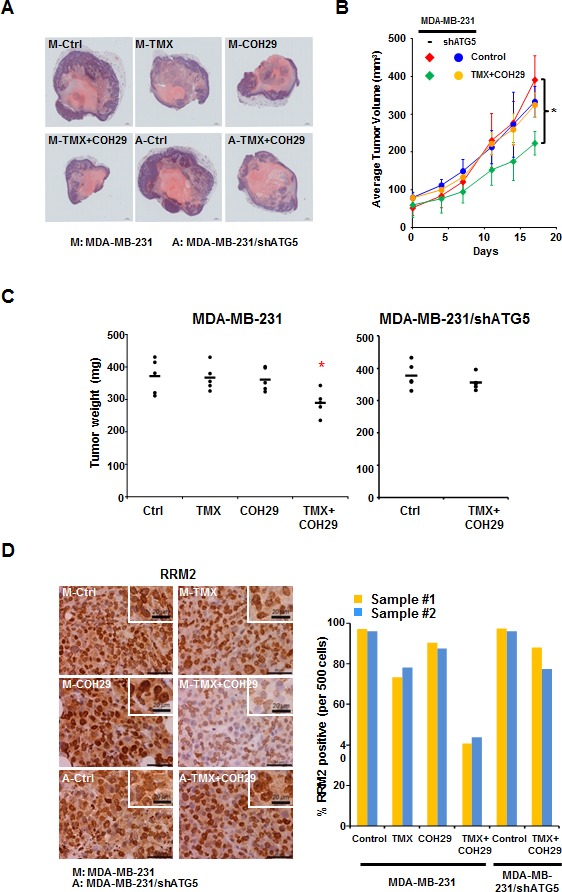
Combination of TMX and COH29 reduces tumor growth *in vivo* **A.** Representative picture of harvested tumors after H&E staining. M: MDA-MB-231 cells; A: MDA-MB-231/shATG cells; T: TMX (80 mg/kg/day, oral); COH: COH29 (30 mg/kg/day, *i.p*.); Ctrl: control; TMX + COH29: tamoxifen (80 mg/kg/day, oral) plus COH29 (30 mg/kg/day, *i.p*.). **B.** Autophagy is required for combined TMX and COH29 to reduce tumor volume. Tumor growth over time is shown as the means ± SD, *n* = 5; _*_: *p* < 0.05. **C.** Autophagy is required for combined TMX and COH29 to reduce tumor weight. The tumor weight at harvest is shown. Each dot represents one mouse and the line indicates the average of the 5 samples. **D.** Combination of TMX and COH29 reduces RRM2 abundance. Representative immunostaining images of RRM2 staining are shown (*left panels*). Scale bar: 10 μm. The enlarged view is shown in the inlet; scale bar: 20 μm. M: MDA-MB-231 cells; A: MDA-MB-231/shATG5 cells; T: TMX (80 mg/kg/day, oral); COH: COH29 (30 mg/kg/day, *i.p*.); Ctrl: control; TMX + COH29: TMX (80 mg/kg/day, oral) plus COH29 (30 mg/kg/day, *i.p*.). RRM2-positive cells per 500 cells were enumerated from each sample (*n* = 2) and the percentage was calculated by comparing to the control (designated as 100%). Each bar in the right panel represents one tumor sample.

Next, we used an orthotopic tumor model and treated the tumor-bearing mice with vehicle, TMX, COH29 or the combination. MDA-MB-231 and MDA-MB-231/shATG5 cells were implanted into the mammary glands of NOD.Cg-*Prkdc*^scid^*Il2rg*^tm1Wjl^/SzJ (NSG) (NOD-SCID) mice to form solid tumors and randomly divided into six groups with 5 mice each for the indicated treatments. Tumors were harvested at day 17 post-treatment and evaluated using hematoxylin and eosin (H&E) staining. Figure 3A shows the differences in tumor size among the groups. The tumor volume was recorded twice a week and plotted as shown in Figure 3B. The autophagy-competent, xenografted tumors showed significantly less growth in the presence of the combined TMX and COH29 treatment, relative to vehicle alone (Figure 3B) and also showed a reduced weight at the time of harvest (Figure 3C). However, consistent with Supplementary Figure S1C, the combined treatment had no effect on tumors from ATG5-knockdown MDA-MB-231 cells (Figure 3B). Furthermore, TMX and COH29 treatment, when delivered individually, did not reduce tumor growth (Figure 3C). Immunohistochemistry staining (IHC) of Ki67, a cellular marker for proliferation, was performed to assess tumor cell growth or not. The staining indicated that less Ki67 signals were detected in xenografted tumor cells from mice receiving the combined treatment of TMX and COH29 (Supplementary Figure S4A), consist with the significant reduction of tumor weights among four treatment groups (Figure 3C). Altogether, our results clearly show that TMX and COH29 have anti-tumor effects both *in vitro* and *in vivo* and suggest that autophagy is required for the combinatorial effect.

Next, the tumor samples were stained with anti-RRM2 and -Ki67 antibodies, respectively (Figure 3D and Supplementary Figure S4A). The percentage of anti-RRM2 positive cells per 500 cells in 2 tumor samples was enumerated. Figure 3D reveals that the nuclear RRM2 abundance was slightly reduced in the TMX-only group, unchanged in the COH29-only group, and markedly decreased in the group treated with TMX+COH29. As expected, the TMX+COH29 treatment only slightly reduced RRM2 abundance in tumors derived from autophagy-impaired MDA-MB-231/shATG5 cells (Figure 3D). These results were validated by Western analyses using protein samples extracted from the indicated tumors (Supplementary Figure S4B). In autophagy-competent MDA-MB-231-derived tumors, RRM1 abundance was not affected by the treatments, whereas RRM2 levels were reduced by combined treatment. Although we were unable to detect LC3-II, the LC3-I level decreased in TMX and became almost undetectable in the group received combined treatment, whereas unlipidated LC3 was notably visible in MDA-MB-231/shATG5-derived samples (Supplementary Figure S4B).

### Tamoxifen treatment enhances COH29-induced DNA damage and mitotic catastrophe

**Figure 4:**
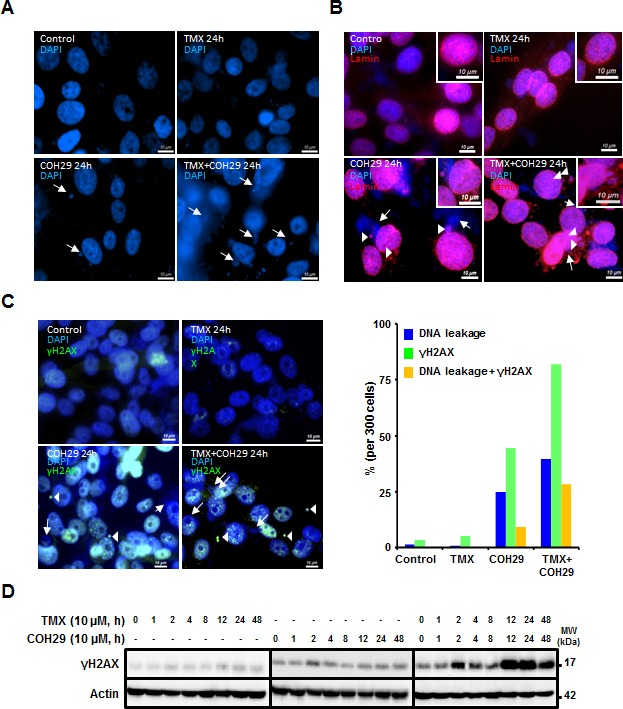
Combination of TMX and COH29 addition induces DNA leakage MDA-MB-231 cells were treated with TMX, COH or the combination for the indicated time periods and stained with DAPI **A.**, anti-lamin **B.** and anti-γH2AX (C, *left panel*). Arrows indicate the leaked DNA. Arrowheads depict the overlap of the leaked DNA with lamin **B.** or γH2AX staining (**C.**, *right panel*). The percentage of DNA leakage, γH2AX positive or containing both phenotype cells is calculated and plotted as a bar graph. scale bar: 10 μm. **D.** Western blot analysis showing the change in γH2AX abundance after TMX, COH and combination treatment.

In order to investigate the mechanism underlying breast cancer cell death by combined treatment with COH29 and TMX, fluorescence microscopy was used to examine the nuclear dynamics. As shown in Figure 4A, one striking feature was that COH29 treatment induced nuclear DNA leakage, as identified by DAPI-positive particles outside the nucleus, and the leaked DNA puncta were notably larger than micronuclei [36]. Furthermore, co-treatment with TMX enhanced the DNA leakage (Figure 4A). Lamins A and C, a structure that surrounds the nucleus, are the scaffolding components of the nuclear envelope in cells [37]. Notable features of this phenotype are a non-disrupted nuclear shape and an intact nuclear membrane, as shown by lamin A/C staining (Figure 4B). Intriguingly, some of the leaked DNAs were within lamin-labeled structures (Figure 4B). Because dNTP depletion is known to induce genomic instability, we next wished to assess the presence of DNA strand breaks. γH2AX is a phosphorylated histone protein that serves as a marker for double strand breaks. The percentage of cells that showed DNA leakage, γH2AX positivity or both was enumerated. Figure 4C shows that COH29-treatment induced DNA leakage and γH2AX positivity, and the COH29+TMX combination greatly enhanced this phenomenon. As expected, TMX treatment alone did not cause the accumulation of γH2AX. Western analyses confirmed a slight increase of γH2AX abundance, which varied over time in COH29-treated cells, (Figure 4D). In contrast, a more abundant γH2AX signal with extended duration was detected in combination-treated cells. It has been shown that autophagy plays a role in DNA damage response by cleaning up damaged DNAs [38-40]. As anticipated, knockdown of ATG5 induced a higher basal level of the basal γH2AX level in MDA-MB-231/shATG5 cells prior to COH29 or TMX treatment, and there was no significant increase of γH2AX level after treatment (Supplementary Figs. S5A and S5B).

**Figure 5:**
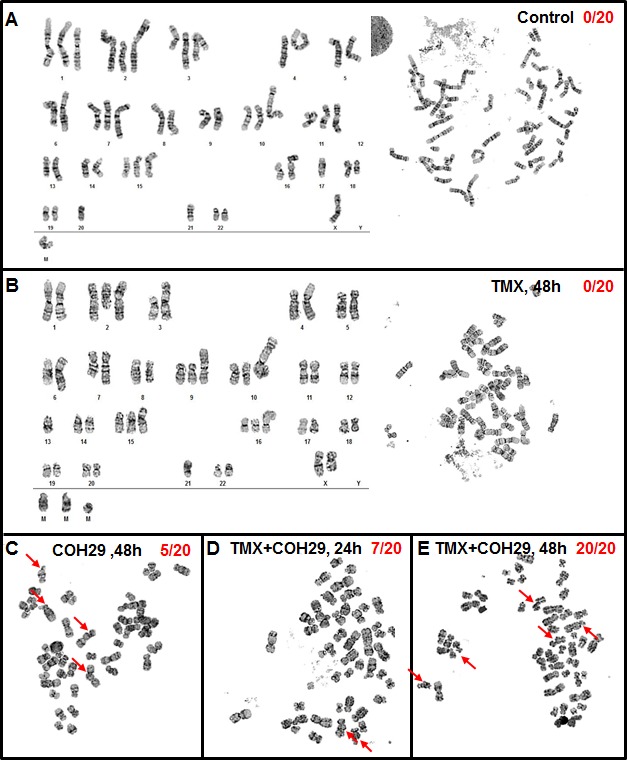
Combination of TMX and COH29 causes chromatin gaps and breakage MDA-MB-231 cells were treated with TMX, COH29 for 48 h or the combination for 24 and 48 h and subjected to cytogenetic analysis. Control **A.**, TMX 48 h **B.**, COH 48 h **C.**, combination 24 h **D.** and 48 h **E.**. Arrows indicate the chromatin gaps and breakage. The numbers shows the quantitation of the breakage as X/Y; X: cells showing chromatin break; Y: total cell number.

In order to investigate the potential genomic instability induced by the combined of COH29 + TMX treatment, MDA-MB-231 cells undergoing various treatments were subjected to cytogenetic analysis (Figure 5). Serving a control, vehicle-treated MDA-MB-231 cells showed an abnormal female karyotype with hyperdiploid and near tetraploid populations. The cells were characterized by structural abnormalities involving chromosomes X, 1, 2, 3, 4, 6, 7, 8, 9, 10, 11, 13, 15, 16, 18, and 21. Clonal numerical gains of chromosomes 1, 2, 3, 10, 11, and 15 were detected, as well as clonal losses of chromosome 21 (Figure 5A). The near tetraploid sideline cells contained duplicate copies of most stemline abnormalities. Using vehicle-treated MDA-MB-231 cells as a reference, we investigated the effects of drug treatment. A subset of the COH29-treated cells (5/20) showed increases in genomic instability, including increases in chromosome and chromatid breaks and gaps, whereas TMX treated cells did not show any increased genomic instability (Figure. 5B and 5C). In the cells co-treated TMX and COH29 for 24 h, the overall chromosomal morphology was significantly worse than that in vehicle-treated cells and chromosomal breakage was apparent in approximately 35% of the cells (Figure 5D). At 48 h after combined treatment, chromosome and chromatid breaks and gaps were observed in almost all cells. In addition, all cells undergoing combined treatment for 48 h were arrested in metaphase, accompanied by shorter chromosome length and loss of chromosomes (Figure 5E). Taken together, these results suggested that COH29 treatment induced DNA damage, resulting in DNA leakage, which was markedly aggravated by combination with TMX.

## DISCUSSION

RR inhibitors, first introduced into clinic near 60 years ago, are widely used in cancer therapy. The use of RR inhibitors as anti-cancer agents has been reviewed by Shao and colleagues [11, 41]. Because rapidly dividing tumor cells have an increased need for dNTPs, they are far more sensitive to the cytotoxic effects of RR inhibition than normal cells. In addition, a growing body of evidence is showing that RRM2 is overexpressed in cancer and is associated with neoplasia, metastatic potential, and poor prognosis in human cancers [42-44].

60 years after the introduction of RR inhibitors into clinical oncology, their mechanism of action in human tumors is becoming increasingly clear. RR is a unique enzyme that utilizes free radicals to drive catalysis in a mechanism that involves components in both the RRM1 and RRM2 subunits [45, 46]. The RRM1 subunit contains the active site and substrate specificity sites, whereas the RRM2 subunit contains the oxygen-linked diferric iron center and tyrosyl radical. Numerous small molecule inhibitors, such as hydroxyurea (HU), gemcitabine (both approved for human cancer clinical use) and triapine [47], interact with the RRM2 subunit and inhibit RR activity. However, these small molecules have problems that limit their use, including lack of specificity for the RRM2 protein, unwanted side effects, and susceptibility to resistance. For instance, HU blocks DNA synthesis by reducing the tyrosyl free radical of the RRM2 subunit to a normal tyrosine residue but is susceptible to resistance. Furthermore, 3-AP (triapine®), which was in human phase II clinical testing, relies on iron chelation to inactivate RR. Data from a phase I trial of 3-AP showed that patients developed side effects such as hypoxia, respiratory distress, and methemoglobulin, due to chelation of iron in the patients' red blood cells [48]. Therefore, approach to improve efficacy of existing RRM2 inhibitor or development of new RRM2 inhibitors are needed [49].

The reciprocal regulation of autophagy and the dNTP pool has been observed and reported in human cancer cells [28]. In this report, we have demonstrated that the ER-independent effect of TMX, autophagy induction, sensitizes ERα-negative MDA-MB-231 cells to COH29, via RRM2 reduction. Studies on the combination of TMX with other agents, such as TRAIL [50] and rapamycin [51], have shown synergistic apoptotic activity. Although the concept of combined TMX with TRAIL therapy is worthwhile, the therapeutic use of TRAIL is limited by concerns over its potential hepatotoxicity [52, 53]. Currently, TMX is associated with depression, endometrial hyperplasia, and Coumadin drug interaction. COH29 causes hematologic toxicity, such as anemia, thrombocytopenia, and leukemia. Theoretically, the side effects from the combination of TMX and COH29 should not exceed their respective adverse effect.

Two different ubiquitin ligases are known to exert tight control over the steady-state levels of RRM2 in the G_1_- and G_2_-phases of cell cycle. During G_1_-phase, APC/CCdh1, a multi-subunit E3 ubiquitin ligase complex, targets RRM2 for degradation [54]. During G_2_-phase, cyclin F controls the degradation of RRM2 [55]. In this report, we found that besides ubiquitin-mediated degradation of RRM2, the abundance of RRM2 was regulated by lysosomal degradation through autophagy induction. As shown in Figures. 1C and 2C, the pretreatment of TMX (to induce autophagy) effectively reduced RRM2 level and sensitized cells to COH29. Moreover, the addition of BafA1, an autophagy inhibitor, blocked the decrease of RRM2 after autophagy induction by TMX, rapamycin and arginine depletion (Figure 1C, Supplementary Figs. S2A and S2C). All these suggest that autophagy-dependent lysosomal degradation plays a critical role in controlling RRM2 level in breast cancer cells. However, the underlying mechanism of how autophagy recognized RRM2 for degradation is still unclear and additional study is warranted. Although, we cannot rule out the possibility that increase in COH29-mediated cytotoxicity is due to increased drug intake via autophagy, we postulated that TMX-induced aggressive autophagy promotes the death of COH29-treated cells. This possibility is supported by the observation that some of the leaked DNAs were within lamin-labeled structures (Figure 4B), consistent with the report by Gonzalez-Suarez et al. that loss-of-lamin A affects the ability of cells to properly repair DNA damage and maintain genome integrity [56].

Anti-mitotic agents have wide-ranging therapeutic potential for the treatment of various types of cancers [57, 58] and there has been a great deal of interest in identifying novel mitotic inhibitors that can overcome the various modes of resistance and promote improved pharmacological profiles [59]. A previous report demonstrated that knockdown RRM2 and the addition of cisplatin induces inter- and intra-strand DNA crosslinks [60]. Also, a recent report from us showed that loss of RRM2b causes chromosomal instability and chromatid breakage in mice [61]. These findings showed that RRM2 plays a crucial role in maintaining chromosomal stability, suggesting that modulation of cellular RRM2 abundance may be important for the induction of mitotic cell death.

We propose that autophagic degradation of a RRM2, the target of COH29, promotes cell death, whereas in other contexts autophagy can protect cancer cells from death [62, 63]. Why does autophagy promote tumor cell survival in some circumstances and death in other circumstances? One possible explanation relates to what autophagy is degrading in each specific circumstance. For example, starvation- or rapamycin-induced autophagy had been shown to decrease RRM2 level and accompanied by a decrease in RNR activity and dNTP pools in human cancer cells [30]. In our study, we showed that COH29 along can induce cell death through inhibiting RR activity, and its cytotoxicity is dramatic enhanced when autophagy is induced by TMX, even though the cells are normally resistant to TMX. In summary, the present findings indicate that combination of TMX and COH29 effectively reduces cell viability in MDA-MB-231 cells via an autophagy-dependent pathway. Furthermore, our data suggest that autophagy induction may serve as an adjuvant anticancer therapy, and that proper combined treatment with an autophagy inducer could synergistically enhance the therapeutic efficacy of a myriad of cancer treatments.

## MATERIALS AND METHODS

### Cell lines, media and chemicals

MDA-MB-231, MDA-MB-231/sh-ATG5 (ATG5 knockdown [5]), MDA-MB-231/GFP-LC3 (stable GFP-LC3 overexpression [5]) and MCF-7 cells were cultured in Dulbecco's modified Eagle's medium (DMEM) (Cellgro, 10-013-CV) containing fetal bovine serum (10%; Gibco, 26140), penicillin (100 U/ml) and streptomycin (100 μg/ml) (Gibco, 15240) at 37°C and 5% CO_2_ in a humidified incubator. T47D cells were cultured in RPMI 1640 medium under similar conditions. MCF-10A cells were maintained in DMEM/F12 media supplied with 5% horse serum, 20 ng/mL EGF, 0.5 mg/mL hydrocortisone, 100 ng/mL cholera toxin, 10 μg/mL insulin (Invitrogen, Carlsbad, CA, USA). Rapamycin (R8781), tamoxifen (T5648), bafilomycin A1 (B1793) and Chloroquine diphosphate salt (C6628) were from Sigma. Recombinant ADI-PEG20 was a gift of Polaris Pharmaceuticals Inc. (San Diego, CA).

### Antibodies

The anti-MAP1LC3-I/II antibody (4599 and 3868), anti-lamin (4777) and anti-caspase-3 antibody (9665) were from Cell Signaling, and the anti-SQSTM1 (p62/SQSTM1) antibody (sc-28359), anti-RRM1 antibody (sc-11733) and anti-RRM2 antibody (sc-10844) were from Santa Cruz. The anti-Ki67 antibody (ab15580) was from Abcam, and the anti-p53R2 antibody (600-401-B67) was from Rockland. The anti-γH2AX antibody (05-636) and anti-actin antibody (MAB1501R) were from Millipore.

### Cytotoxicity and apoptosis assays

Many cytotoxicity assays are dependent on mitochondrial function and can generate artifacts if mitochondrial function is impaired without cell death. Therefor, ACP assays, which measures cellular acid phosphatase activity, were used to avoid the effects of mitochondria dysfunction. Between 2,500 and 5,000 cells were seeded onto 96-well plates. Cells were treated with TMX, COH or the combination at the indicated concentrations for 72 h. At the end of the time period, cells were washed twice with PBS then incubated at 37°C for 30 min with 100 μl of pNPP solution composed of pNPP (5 mM) in a buffer containing sodium acetate (0.1 M) and Triton X-100 (0.1% (v/v), pH 5.5). The reaction was terminated by adding NaOH (1 N, 10 μl), and the absorbance was measured at 410 nm using a microplate reader. The interaction between drug combinations was analyzed using the Calcusyn software program (Biosoft, Cambridge, UK) to determine if the combination was antagonistic, additive or synergistic. This program is based on the Chou-Talalay method to calculate a CI, and CI values below 1 indicate synergistic effect. The CIs were determined from cell viability ACP assays as the fraction of cells killed by individual drugs, or combination of drugs, compared to vehicle-treated cells. For the apoptosis assay, cells were treated with COH29 and TMX for the indicated time periods. After trypsinization, cells were washed twice with cold PBS and collected by centrifugation at 1000 rpm. Cells were then resuspended in 1 × Binding Buffer at a concentration of 1 × 10^6^ cells/ml and 100 μl of the suspension (1 × 10^5^ cells) was transferred to a polystyrene round-bottom tube. Cells were then stained with FITC-conjugated Annexin V (4 μl) and propidium iodide (PI) (50 μg/ml, 5 μl). The mixture was gently vortexed and incubated for 15 min at RT and 1 × Binding Buffer (400 μl) was added to each tube before analyzing by flow cytometry.

### Whole-cell Extracts and Immunoblotting

Cells treated with TMX, COH or combination treatment were harvested at the end of incubation period and lysed on ice for 30 min in RIPA buffer (Cell Signaling, #9806) containing a complete protease inhibitor cocktail (Roche, 11836145001) and PhosSTOP (Roche, 04906837001). The Qproteome Mammalian Protein Prep Kit (Qiagen) was used to extract protein from harvested tumors. The protein concentrations were determined using a Bio-Rad Protein Assay Kit (Bio-Rad, 500-0001). Approximately 40 μg of protein was mixed with an equal volume of 2 × SDS loading buffer, boiled for 5 min, then separated by Tris-glycine SDS-PAGE and transferred to PVDF membranes. The membranes were blocked with 5% nonfat milk in PBST (PBS containing 0.05% Tween 20) and incubated with primary antibodies at 4°C overnight. The membranes were then washed with three times with PBST for 10 min, and incubated with HRP (horseradish peroxidase)-labeled secondary antibodies for 2 h at RT. Immunoblots were visualized using the VersaDoc 5000 imaging system (Bio-Rad) and processed using quantity one (Bio-Rad).

### Isolating dNTPs

1 × 10^6^ cell pellets were harvested and added to 100 μl of 15% trichloroacetic acid. Alternatively, 50 mg of tumor samples were added to 100 μl of 15% trichloroacetic acid. The solution was mixed and homogenized using a TissueLyser (Qiagen) for 1 min then kept on ice for 10 min. Then the mixture was centrifuged for 5min, and the supernatant fractions were saved and extracted with 2 × 50 μl aliquots of 1,1,2-trichlorotrifluoroethane/trioctylamine (55:45; Sigma-Aldrich,270369, and T81000). After each centrifugation, the supernatant was saved. 5 μl aliquots of each sample were used to check the dATP, dCTP, dGTP, and dTTP concentrations. The remainder was stored at −80°C for further assays.

### dNTP pool assay

This assay was conducted according to the method of Sherman and Fyfe. The total reaction volume was 50 μl. The reaction mixture contained 50 mM of Tris-HCl (pH 7.5), 10 mM of MgCl2, 5 mM of DTT, 0.25 mM of template/primer, 1.25 mM of [^3^H]dATP (for dCTP, dGTP, and dTTP assay) or [^3^H]dTTP (for dATP assay), and 0.2 units of Sequenase (2.0) (Affymetrix, 70775Y). After reacting at room temperature (RT) for 20 min, 40 μl of the aliquots were applied to circular Whatman DE81 ion exchange paper (GE Healthcare Life Sciences, 3658-325). The papers were dried, then washed 3 × 10 min with 5% Na_2_HPO_4_, and rinsed once with distilled water and once more with 95% ethanol. After each paper was dried and deposited in a small test tube, and 5 ml of Ecoscint A was added to each tube. Tritium-labeled dNTPs were counted in a liquid scintillation counter and compared to a standard sample prepared in the presence of 0, 0.25, 0.50, 0.75 and 1.0 pmol/ml of dATP, dTTP, dGTP, and dCTP each.

### Microscopy

MDA-MB-231/GFP-LC3 cells were cultured in six-well plates with cover slips at a density of 1 × 10^5^ cells/well, and then treated with TMX, COH or the combination for the indicated time points. After washing with PBS, the cover slips were mounted over a microscope slide in Prolong anti-fade reagent that contained DAPI (Life Technologies, P-36931), and examined using a Olympus AX70 upright microscope (Olympus).

### Xenograft studies

For tumorigenesis, 5 × 10^6^ MDA-MB-231 and MDA-MB-231/sh-ATG5 cells (resuspended in 100 μl serum-free DMEM media) were used for orthotopic implantation of six-week-old female NOD.Cg-*Prkdc*^scid^*Il2rg*^tm1Wjl^/SzJ (NSG) (NOD-SCID) mice. Mice were randomized at day 10 after tumor inoculation and received vehicle, an approximate total dose of 30 mg/kg of COH29, 80 mg/kg of TMX, or the combination therapy daily for 17 days (n = 5 per group). The tumor volume was measured twice every week. Tumors were harvested for weight measurement and H&E, RRM2 and Ki67 staining. All animal experiments were approved by the institutional animal care and use committee at City of Hope.

### Immunohistochemistry (IHC)

Ki67 and RRM2 protein levels in the tumor samples harvest from mouse were assessed by IHC using anti-Ki67 (1:100 dilution) and anti-RRM2 antibodies (1:75 dilution). Briefly, after de-paraffinization, endogenous peroxidase activity was blocked by pre-treatment with 3% H_2_O_2_. The slides were incubated with normal goat serum for 20 min at RT to block non-specific signal, then incubated with the primary antibody for 20 min at RT. The slides were then incubated with polymer horseradish peroxidase-labeled secondary antibodies for 30 min at RT, then 3,3-Diaminobenzidine (DAB)-treated (0.05 g DAB and 100 ml 30% H_2_O_2_ in 100 ml PBS) for 5 and 10 min, respectively.

### Statistical analysis

Data analysis was performed using GraphPad Prism 5.0 software or Microsoft excel. Each cell biology experiment was performed in triplicate to obtain representative means and images. Statistical significant was set as *p* < 0.05, two-tailed.

## SUPPLEMENTARY MATERIAL FIGURES


