# Supplementary material for: Autophagy induction causes a synthetic lethal sensitization to ribonucleotide reductase inhibition in breast cancer cells
Source: Oncotarget. 2015 Dec 9;7(2):1984–99. doi: 10.18632/oncotarget.6539 (PMC4811511; doi:10.18632/oncotarget.6539)
Supplement: Supplementary file 1 [file oncotarget-07-1984-s001.pdf]

Autophagy induction causes a synthetic lethal sensitization to ribonucleotide reductase inhibition in breast cancer cells

Supplementary Material

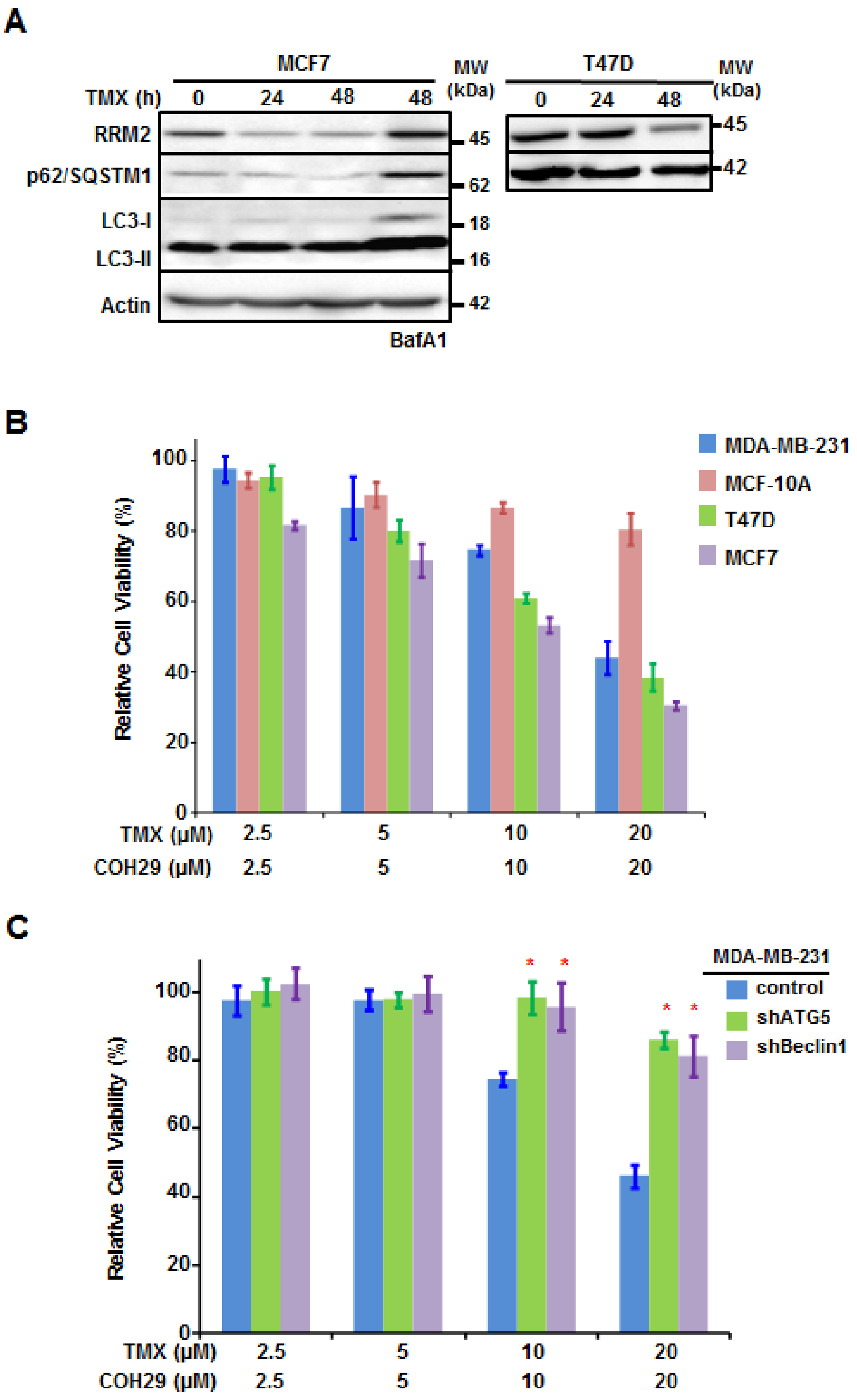

Supplementary Figure S1: (**A**) TMX decreases RRM2 expression and induces autophagy in

MCF7 and T47D breast cancer cells. (**B**) MCF-10A cells are resistant to the combination of TMX and COH29. Relative cell viability in the MDA-MB-231, T47D MCF7, and MCF-10A cells treated with the combination of TMX and COH29 is shown. No treatment controls are defined as 100% viable. Results are shown as means ± SD; n = 3. (C) ATG5 or BECLIN1 is indispensable for the cytotoxicity of the combination of TMX and COH29. Relative cell viability in MDA-MB-231, MDA-MB-231/shATG5, and MDA-MB-231/shBECLIN1 cells treated with the combination of TMX and COH29 is shown. No treatment controls are defined as 100% viable. Results are shown as means ± SD; n = 3.

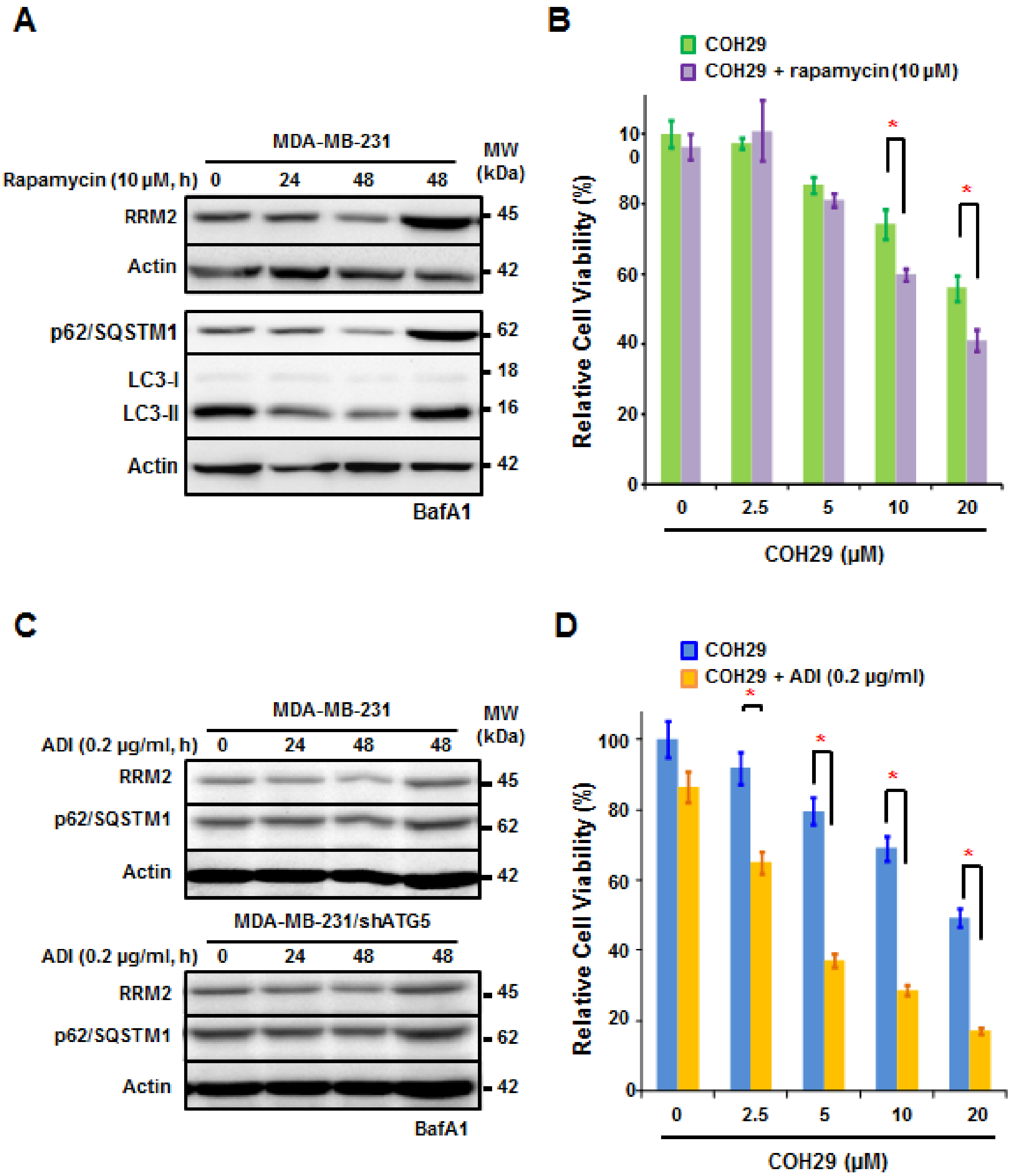

Supplementary Figure S2: (**A**, **C**) Western Blot analyses showing treatment of rapamycin (10 μM) or ADI-PEG20 (ADI, 0.2 μg/ml) induces autophagy and decreases RRM2 expression in MDA-MB-231 cells, but had no effect on MDA-MB-231/shATG5 cells. (**B**, **D**) Relative cell viability in MDA-MB-231 cells treated with the combination of rapamycin (**B**) or ADI-PEG20 (**D**) with COH29. No treatment controls are defined as 100% viable. Results are shown as means ± SD; n = 3.

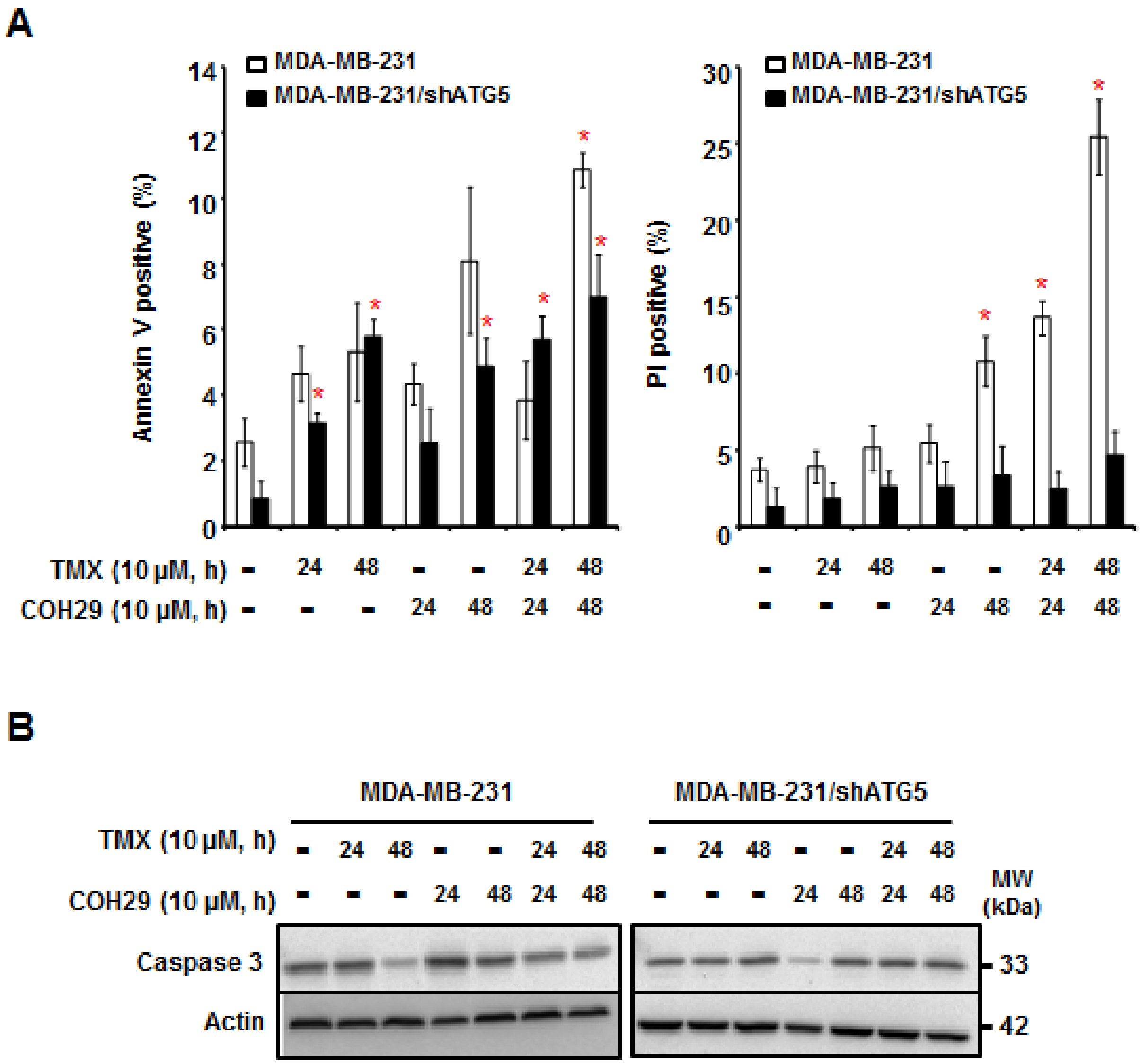

Supplementary Figure S3: (**A**) MDA-MB-231 cells and MDA-MB-231/shATG5 cells were treated with TMX, COH or combination for indicated time periods and stained with Annexin V and PI for apoptosis analysis. The positively stained population is compared to the no staining control and the percentage is plotted as a bar graph showing Annexin V on the left and PI on the right panel. Results are shown as means ± SD; n = 3; *: $p < 0.05$. (**B**) Western Blot analyses showing the change in caspase 3 abundance after TMX, COH and combination treatment.

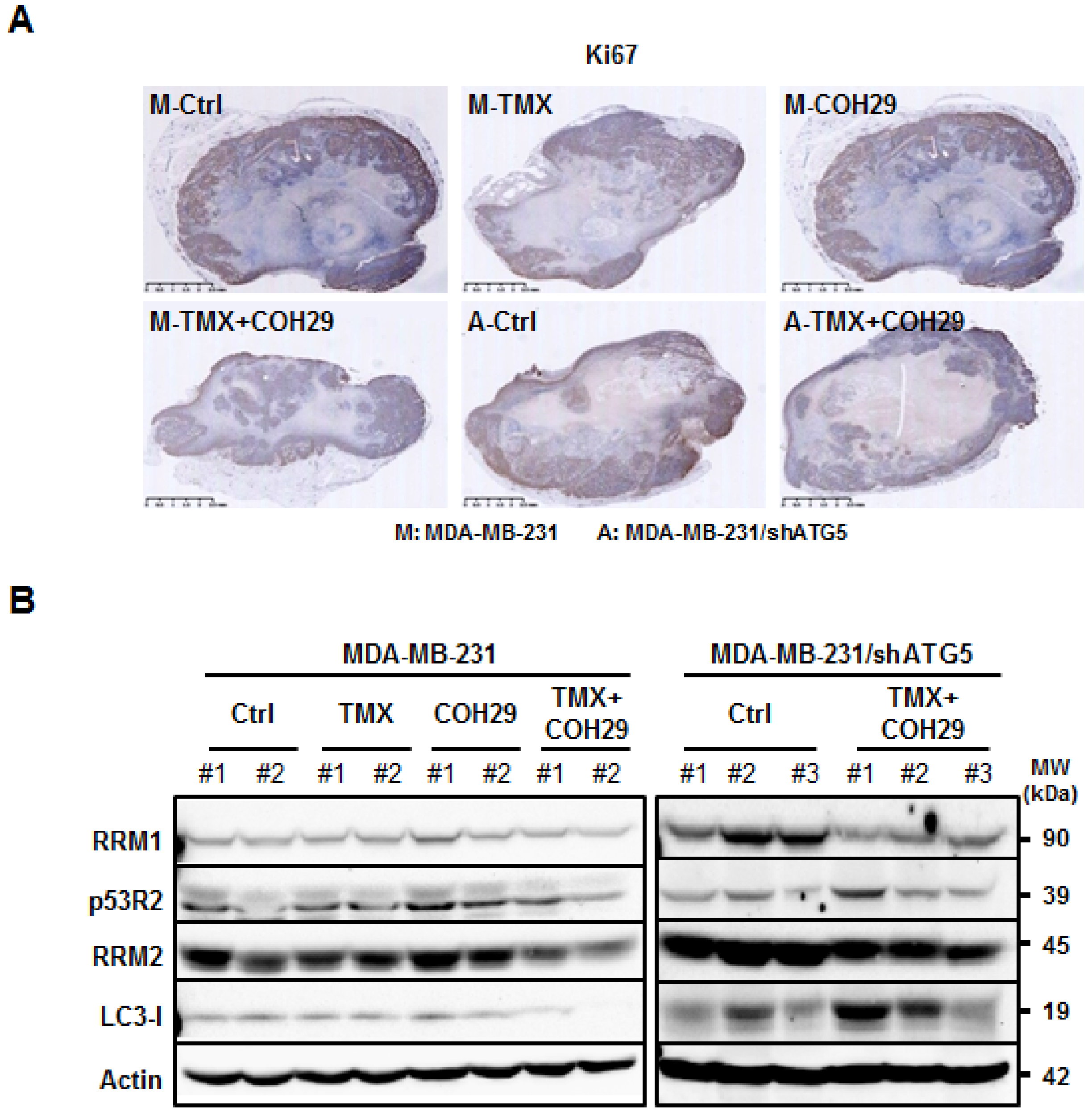

Supplementary Figure S4: (**A**) Representative tiling picture of harvested tumors after Ki67 staining. (**B**) Western blot analysis showing the changes in RRM1, p53R2, RRM2 and LC-3 abundance in harvested tumor samples. M: MDA-MB-231 cells; A: MDA-MB-231/shATG5 cells; Ctrl: control; T: TMX; COH: COH29.

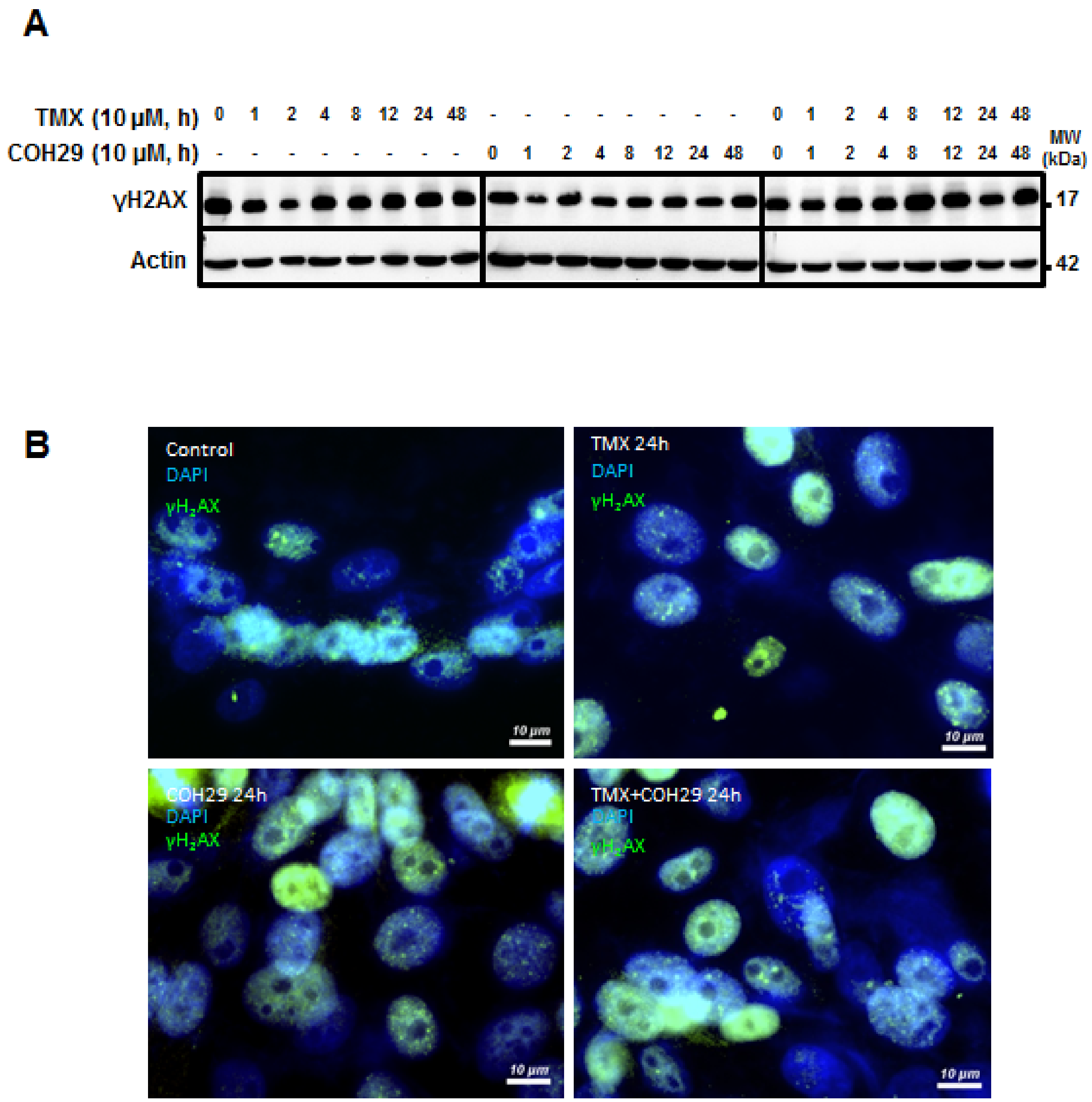

Supplementary Figure S5: (**A**) Western blot analysis showing the change in γH2AX abundance in MDA-MB-231/shATG5 cells treated with TMX, COH or combination for indicated time periods. (**B**) MDA-MB-231/shATG5 cells were treated with TMX, COH or the combination for the indicated time periods and stained with DAPI and anti-γH2AX antibody. Scale bar: 10 µm.
